# Predictors of outcome after endovascular treatment for tandem occlusions: a single center retrospective analysis

**DOI:** 10.1186/s12883-023-03127-4

**Published:** 2023-02-27

**Authors:** Brian Anthony B. Enriquez, Terje Nome, Cecilie G. Nome, Bjørn Tennøe, Christian G. Lund, Mona K. Beyer, Mona Skjelland, Anne Hege Aamodt

**Affiliations:** 1 Department of Neurology, Oslo University Hospital, Oslo, Norway; 2 Division of Radiology and Nuclear Medicine, Oslo University Hospital, Oslo, Norway; 3 Division of Anatomy, Department of Molecular Medicine, Institute of Basic Medical Sciences, Faculty of Medicine, University of Oslo, Oslo, Norway; 4 Institute of Clinical Medicine, University of Oslo, Oslo, Norway

**Keywords:** Tandem occlusions, Endovascular therapy, Thrombectomy, Stenting, Diabetes, Symptomatic intracranial hemorrhage

## Abstract

**Background:**

The endovascular treatment procedure in tandem occlusions (TO) is complex compared to single occlusion (SO) and optimal management remains uncertain. The aim of this study was to identify clinical and procedural factors that may be associated to efficacy and safety in the management of TO and compare functional outcome in TO and SO stroke patients.

**Methods:**

This is a retrospective single center study of medium (MeVO) and large vessel occlusion (LVO) of the anterior circulation. Clinical, imaging, and interventional data were analyzed to identify predictive factors for symptomatic intracranial hemorrhage (sICH) and functional outcome after endovascular treatment (EVT) in TO. Functional outcome in TO and SO patients was compared.

**Results:**

Of 662 anterior circulation stroke patients with MeVO and LVO stroke, 90 (14%) had TO. Stenting was performed in 73 (81%) of TO patients. Stent thromboses occurred in 8 (11%) patients. Successful reperfusion with modified thrombolysis in cerebral infarction (mTICI) ≥ 2b was achieved in 82 (91%). SICH occurred in seven (8%). The strongest predictors for sICH were diabetes mellitus and number of stent retriever passes. Good functional clinical outcome (mRS ≤ 2) at 90-day follow up was similar in TO and SO patients (58% vs 59% respectively). General anesthesia (GA) was associated with good functional outcome whereas hemorrhage in the infarcted tissue, lower mTICI score and history of smoking were associated with poor outcome.

**Conclusions:**

The risk of sICH was increased in patients with diabetes mellitus and those with extra stent-retriever attempts. Functional clinical outcomes in patients with TO were comparable to patients with SO.

## Background

Tandem occlusions (TO) are defined as intracranial vessel occlusion with concomitant high-grade stenosis or occlusion of the ipsilateral cervical internal carotid artery (cICA) and occur in around 15% of patients receiving endovascular treatment (EVT) in the anterior circulation [1–3]. The EVT procedure in TO is more complex than in single occlusions (SO) as it necessitates treatment of two lesions, possible use of stent and consequently, an early initiation of antiplatelet therapy. The optimal management therefore remains uncertain [4].

To date, no randomized controlled trial addressing the management of TO have been completed. Large meta-analyses suggest an advantage of acute cICA stenting during EVT for TO patients [5, 6]. Furthermore, prioritizing treatment of the intracranial occlusion before the extracranial lesion may yield higher reperfusion and better functional outcome [7]. The current 2019 AHA/ASA guidelines on endovascular treatment of TO recommend EVT with recanalization of both extracranial and intracranial occlusions (class IIb, level B-R) [8]. However, the technical approach, timing in the treatment of cICA and medical management are still unsettled [8, 9]. More information is needed to increase the chances of better outcome and reduce serious adverse events such as symptomatic intracranial hemorrhage (sICH), embolization in new territories and stent thrombosis.

The aim of this study was to compare functional outcome 90 days after treatment of TO and SO stroke patients and identify factors that may be associated to efficacy and safety in the management of TO.

## Methods

This study is based on data from the Oslo Stroke Reperfusion Study (OSCAR) – a register of consecutive stroke patients treated with EVT at Oslo University Hospital, which is a highly specialized regional university hospital covering 3.1 million inhabitants during the study period. Data on patient demographics, premorbid status, and clinical parameters including, National Institutes of Health Stroke Scale (NIHSS) score upon admission and discharge, pre-interventional radiological studies, bridging therapy, procedural variables, and complications as well as functional outcome at 90-day follow-up were registered.

The present study included SO and TO patients treated with EVT from January 2017 to October 2020. TO was defined as concomitant intracranial occlusion in the anterior circulation and severe stenosis or occlusion of the cICA. Baseline characteristics, comorbidity, safety measures and clinical outcome in TO and SO patients were compared. The influence of clinical and procedural factors on safety and functional outcomes in TO patients were evaluated. Detailed data acquisition of the radiologic and EVT parameters in TO patients was done retrospectively. The regional ethics committee approved the study and written consent were obtained either from the patient or their legal authorized representative.

### Imaging assessment

CT scans with angiography with or without perfusion were utilized to screen patients with stroke symptoms who may be candidates for reperfusion therapies. Eligible patients for thrombolysis received treatment before transfer to our hospital for EVT or at our institution for those directly admitted and for in-hospital stroke patients. Additional imaging with brain MRI or updated CT scans were done upon arrival in cases with long transport time, altered clinical presentation, wake-up stroke, stroke of unknown onset, and when primary imaging shows poor collaterals.

The recanalization procedure was performed either in general anesthesia (GA) or under conscious sedation (CS) based on the clinician’s discretion. Involvement of distal intracranial occlusions, increased likelihood of stenting or technical difficulty, and restless, agitated, or uncooperative patients were more likely to receive GA. Cerebral digital subtraction angiography and EVT was performed via femoral access. EVT of the intracranial occlusion was performed by aspiration alone, with stent retriever and distal aspiration or in combination. The cICA lesion was treated with stenting and/or percutaneous transluminal angioplasty. Method for treatment, the order of treatment for intracranial and extracranial lesions, and the type of stent used was decided by the interventional neuroradiologist.

When a permanent stent was placed, all patients were given a loading dose of 300 mg Acetylsalicylic acid. Clopidogrel or Ticagrelor was administered either perioperatively or within the first 24 h, not exceeding 300 or 180 mg respectively, in total cumulative dose. When the risk of stent thrombosis was considered highly probable, monotherapy with glycoprotein 2b/3a inhibitor, Eptifibatide, was given as an alternative in bolus of 90 mcg/kg dose followed by infusion at a rate of 2 ug/kg/min. After ruling out hemorrhagic complications on follow up imaging, double antiplatelet therapy with Acetylsalicylic acid 75 mg and Clopidogrel 75 mg or Ticagrelor 60 mg twice daily was started in all patients. Roadsaver® Carotid stent (Terumo, Japan) and Precise stent (Cordis, USA) were utilized in stenting the proximal lesion. Blood pressure was adjusted based on factors such as final recanalization status, core infarction size and location, thrombolysis, and stent utilization.

The modified thrombolysis in cerebral infarction (mTICI) score was rated based on the final digital subtraction angiogram [10, 11]. Scoring was evaluated by two experienced, independent interventional neuroradiologists blinded to clinical information and outcome. Disagreements were resolved by consensus. Successful reperfusion was defined as mTICI 2b, 2c or 3. MRI was obtained using Magnetom Aera 1.5 T or Magnetom Avanto Fit 1.5 T scanner (Siemens Healthcare, Erlangen, Germany). The acquisition time for the diffusion weighted imaging (DWI) protocol was 1:54 – 2:01 with the following parameters: TE 5900–6300 ms, TR 89 ms, slice thickness 5 mm and 30% distant factor. Patients underwent routine follow-up MRI or CT scan within 24 h after endovascular therapy. Ischemic lesions were assessed using the Alberta stroke program early CT (ASPECT) score. Infarct volume was manually outlined based on DWI using B-value 1000 s/mm2 in combination with ADC map and calculated by multiplying the outlined slice volume with the slice thickness and interslice gap. Intracranial hemorrhage was determined using the Heidelberg bleeding classification (HBC) [12]. Worsening of NIHSS score ≥ 4 secondary to hemorrhage was considered as symptomatic intracranial hemorrhage (sICH) [13].

### Statistical analysis

Statistical analyses were performed using SPSS (V.26.0). Continuous variables were expressed as median (interquartile range, IQR) or mean (standard deviation, SD) according to data distribution. Categorical variables were expressed as frequency (percentage) and bivariate analyses were performed using the χ^2^ or Fisher’s exact tests. Prior to regression analyses for functional outcome as the dependent variable, multi-collinearity between possible predictor variables was assessed by the variance inflation factor (VIF), with the tolerance value set at < 2. Multiple logistic regression analyses were performed using variables having a level of significance *p* < 0.05 in the bivariate analyses. A univariate logistic regression was done, with sICH being the dependent variable and continuous variables as independent variables when linearity and a *p* < 0.10 in bivariate analysis was fulfilled.

## Results

### Patients and procedures

In the study period, a total of 745 patients underwent EVT for acute stroke, comprising 662 anterior circulation medium vessel (MeVO) or large vessel occlusion (LVO) stroke who were included in the study. Direct admission and in-hospital stroke comprised of 25 patients, 23 (4%) in SO and 2 (2%) in TO, while the rest were transfers from other hospitals.

**Fig. 1:**
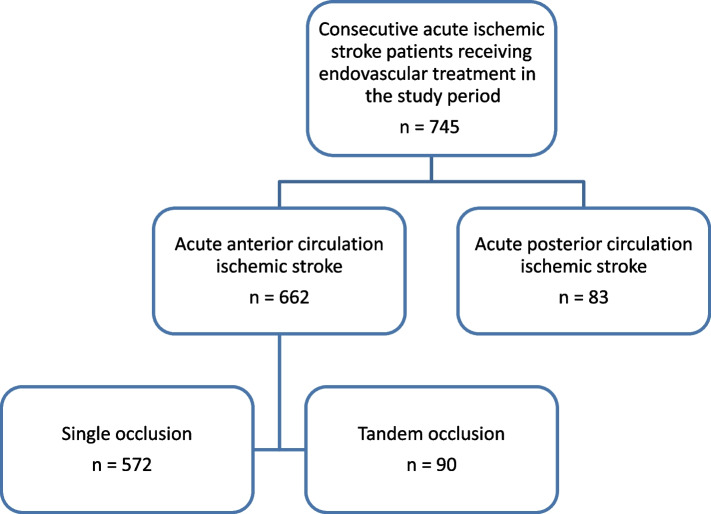
Study flowchart

Ninety (14%) patients met the criteria for TO (Fig. 1). Seven (8%) patients were primarily observed first and had a delayed EVT upon clinical deterioration. Five (5.5%) patients arrived with no available imaging while 28 (31%) had imaging done at the referring hospital and went straight to the angio suite. In total, 50 (56%) patients had repeat imaging on arrival, of which 15 (30%) had unknown stroke onset or wake-up stroke, 15 (30%) patients had more than 2 h lapse since primary imaging was taken, 3 (6%) had ictus > 6 h earlier and 3 (6%) had NIHSS < 4. A distal migration of the intracranial thrombus on repeat imaging was observed in 10 (11%) patients, 6 (60%) of whom received bridging therapy. Perfusion imaging using CT or MR was done in a total of 64 (71%) patients in the TO group.

### Tandem occlusion versus single occlusion

**Fig. 2:**
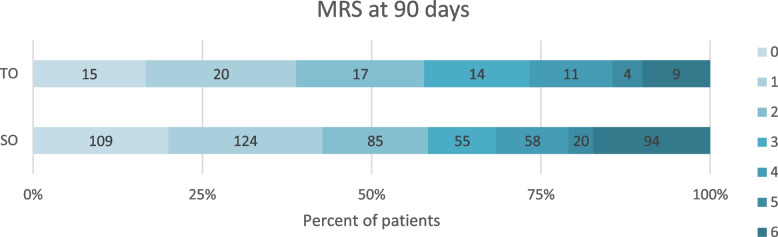
Functional outcome at 90-day follow-up assessed by modified Rankin Scale (mRS) in acute anterior circulation stroke patients with tandem (TO) and single (SO) occlusions undergoing endovascular treatment

Compared to the SO group, the TO group were younger (median age 68 vs 72 years, *p* ≤ 0.001) and predominantly male (68% vs 50%, *p* = 0.001). Furthermore, comorbidity with heart failure, previous transient ischemic attack TIA or stroke as well as atrial fibrillation and consequently, use of anticoagulation was less prevalent in the TO group (Table 1). Stroke severity on admission, use of intravenous thrombolysis and time from stroke onset to puncture did not differ between the groups whereas median procedure time was significantly longer in the TO group. Good functional outcome, defined as mRS ≤ 2 at 90-day follow-up, was achieved in 52 (58%) of the 90 TO patients and 318 (56%) of the SO patients (Fig. 2).

**Table 1:** Baseline characteristics in acute anterior circulation stroke patients with tandem (TO) and single (SO) occlusions undergoing endovascular treatment

Characteristic	Tandem occlusion (*N* = 90)	Single occlusion (*N* = 572)	*P* value
Age, years^a^	68 (56–74)	72 (63–81)	** < 0.001**
Gender (men)	61 (68)	284 (50)	**0.001**
Arterial hypertension	48 (53)	277 (49)	0.394
Diabetes mellitus	12 (13)	81 (14)	0.805
Current or previous tobacco use	48 (51)	201 (65)	0.091
Heart failure	5 (6)	103 (18)	**0.002**
Atrial fibrillation	10 (11)	271 (48)	< 0.001
Previous stroke/TIA	10 (11)	112 (20)	0.045
Hyperlipidemia	17 (19)	88 (16)	0.412
Antiplatelets	26 (29)	175 (31)	0.756
Anticoagulation	6 (7)	144 (25)	** < 0.001**
Known onset	59 (66)	406 (71)	0.282
NIHSS score prior to EVT^a^	15 (10–19)	13 (8–18)	0.089
IV-tPA	51 (57)	320 (56)	0.946
MR/CT taken before thrombectomy	63 (70)	374 (66)	0.632
Time from stroke onset/recognition to puncture, min^a^	248 (181–340)	240 (184–305)	0.364
Time from stroke onset/recognition to recanalization, min^a^	319 (240–400)	285 (226–366)	**0.041**
Procedure time, min (IQR)^a^	95 (75–136)	58 (40–85)	** < 0.001**

*TIA* transient ischemic attack, *NIHSS* national institute of health stroke scale, *EVT* endovascular treatment, *IV-tPA* intravenous tissue-type plasminogen activator

Values are expressed as number (%) or ^a^median (IQR)

### Tandem occlusion

A total of 72 (80%) TO patients had a combination of extracranial carotid lesion and LVO whereas 18 (20%) had MeVO based on the initial CT scan. Internal carotid artery atherosclerosis was present in 71 (79%) patients while 19 (21%) had carotid dissection. Stent was employed in a total of 73 (81%) comprised of 39 (53%) using Roadsaver alone, 32 (44%) patients with Precise carotid stent, and 2 (3%) using both Roadsaver and Precise. A single stent was employed in 50 (69%) patients, 22 (30%) patients with two stents and 1 (1%) patient was treated with 3 stents. Antiplatelet therapy with Acetylsalicylic acid and Clopidogrel were given to 66 (90%) while 7 (10%) received Eptifibatide. Successful reperfusion of MeVO or LVO with at least TICI 2b was achieved in 82 (91%) patients. Stent thrombosis occurred in 8 patients (11%) on follow-up imaging. Six of which received thrombolysis though no significant association was found (*p* = 0.264). No stent thrombosis occurred in all 7 patients who received Eptifibatide, though not significant (*p* = 0.999). The median time from recanalization to stent thrombosis detection was 24 h and 38 min. Furthermore, no association was found between stent thrombosis and prior use of antiplatelet (*p* = 0.216), anticoagulation (*p* = 1.0), number of stents used (*p* = 0.155), platelet count (*p* = 0.972), cancer (*p* = 0.586), diabetes (*p* = 1.0) and smoking (*p* = 0.438). Seven of 8 stent thrombosis occurred in patients who were implanted dual layer stent (Roadsaver) which was significant (*p* = 0.049).

### Hemorrhagic complications in tandem occlusion

Hemorrhage on control imaging for TO were found in 50 (56%) patients, of which 8 (8.8%) patients had both intra- and extra-axial hemorrhage (HBC 1–3). Distal aspiration alone was done in 2 (4%) patients, 47 (94%) treated using stent retriever with distal aspiration, and 1 (2%) patient had proximal clot disintegration with distal migration after stenting of the internal carotid artery. Symptomatic intracranial hemorrhage in the TO group occurred in 7 (7.8%) patients, including 6 (86%) with intra-axial hemorrhage (HBC 1C-2) with or without extra-axial hemorrhage and 1(14%) had solely subarachnoid hemorrhage. All were treated using both stent retriever with distal aspiration thrombectomy. Comparison of basic characteristics, comorbidity, imaging, and treatment including procedural techniques are demonstrated in Table 2. Diabetes mellitus was associated with considerably increased sICH risk (OR 6.2; 95% CI, 1.2–32.2; *P* = 0.047). The risk of sICH also increased for every extra attempt of stent retriever passes made (OR 1.7; 95% CI, 1.0–2.6; *P* = 0.032). Among the patients who received stents, one treated with Eptifibatide had symptomatic bleeding (*P* = 0.522).

**Table 2:** Comparison of variables between symptomatic and no symptomatic intracranial hemorrhage in the tandem occlusion cohort

Characteristic	sICH (*N* = 7)	No sICH (*N* = 83)	*P* value
Age, years^a^	57 (52–77)	68 (57–74)	0.582
Gender (men)	6 (86)	55 (66)	0.421
Arterial hypertension	6 (86)	42 (51)	0.166
Diabetes mellitus	3 (43)	9 (11)	**0.047**
Current or previous tobacco use (*n* = 73)	4 (80)	44 (65)	0.066
Heart failure	1 (14)	4 (5)	0.339
Atrial fibrillation	1 (14)	9 (11)	0.575
Previous stroke/TIA	1 (14)	9 (11)	0.575
Hyperlipidemia	2 (29)	15 (18)	0.613
Antiplatelets	4 (57)	22 (27)	0.186
Anticoagulation	0	5 (6)	1.00
NIHSS score prior to EVT^**^	16.57 ± 5.7	13.86 ± 6.6	0.296
IV-tPA	4 (57)	46 (57)	1.00
DWI ASPECTS ≥ 6 (*N* = 62)	3 (50)	37 (66)	0.657
DWI volume (ml) prior to EVT^a^ (*N* = 63)	27 (11.4–90.2)	16 (8.7–35.7)	0.454
MRI SWI microbleeds prior to EVT^a^ (*N* = 62)	0 (0–2.25)	0 (0–0)	0.502
Time from stroke onset/recognition to puncture, min^a^	365 (212–875)	240 (180–321)	0.086
Time from stroke onset/recognition to reperfusion, min^a^	448 (238–745)	318 (239–377)	0.221
Procedure time, min^a^	129 (48–210)	95 (75–135)	0.772
General anesthesia	3 (43)	58 (70)	0.206
Stent employed (*N* = 73)	7(100)	66 (80)	0.339
Reocclusion of the carotid stent	1 (20)	7 (8)	0.410
Intracranial lesion vs stent first			0.703
Perioperative antiplatelet therapy			0.522
Type of stent			0.566
Thrombectomy technique			
Stent retriever with distal aspiration	7 (100)	65 (78)	0.791
mTICI ≥ 2b	6 (86)	76 (92)	0.491
MEVO involvement	1 (14)	17 (21)	1.00
Changed occlusion location	1 (14)	9 (11)	0.579
No. of stents placed^a^	2 (1–2)	1 (1–1)	0.085
Stent retriever passes^a^	3 (1–5)	1 (1–2)	**0.032**

*sICH* symptomatic intracranial hemorrhage, *TIA* transient ischemic attack, *NIHSS* national institute of health stroke scale, *EVT* endovascular treatment, *IV-tPA* intravenous tissue-type plasminogen activator, *DWI* diffusion weighted imaging, *ASPECTs* Alberta stroke programme early CT score, *mTICI* modified thrombolysis in cerebral infarction, *MEVO* medium vessel occlusion

Values are expressed as number (%), ^a^median (Interquartile range, IQR) or ^**^mean (standard deviation, SD)

### 90-day functional outcome in tandem occlusion

Increasing NIHSS score on admission, time from stroke onset to treatment, smoking history and hemorrhagic transformation in the infarcted tissue were associated with worse outcome in TO patients (Table 3). Use of GA was associated with good outcome. Obviously, DWI volume after EVT and change in DWI volume before and after EVT as well as mTICI correlated with functional outcome. In multivariate analysis both presence of hemorrhage (OR 0.5; 95% CI, 0.3–0.8; *p* = 0.005) and lower mTICI scores (OR 2.0; 95% CI, 1.2–3.4; *p* = 0.005) were the strongest negative predictors of unfavorable functional outcome at 90-day follow-up. In the stented patients, neither the sequence in treating the intracranial and extracranial occlusion nor the carotid pathology showed significant association with outcome. Repeated imaging was associated with poor outcome. Further analysis of patients with or without repeated imaging showed significant difference in age, NIHSS and median door to puncture time (47 vs. 64 min, *p* = 0.001). However, there was no significant association between door to puncture time and mRS at 90 days.

**Table 3:** Comparison of variables between patients with good and poor outcome in the tandem occlusion cohort

Characteristic	Good outcome (*N* = 52)	Poor outcome (*N* = 38)	*P* value
Age, years^b^	64 ± 11.8	68 ± 10.9	**0.049**
Gender (men)	35 (67)	26 (68)	0.911
Arterial hypertension	25 (48)	23 (61)	0.242
Diabetes mellitus	5 (10)	7 (18)	0.225
Current or previous tobacco use	26 (57)	22 (81)	**0.030**
Heart failure	1 (2)	4 (11)	0.158
Atrial fibrillation	4 (8)	6 (16)	0.312
Previous stroke/TIA	5 (10)	5 (13)	0.737
Hyperlipidemia	8 (15)	9 (24)	0.320
Antiplatelets	12 (23)	14 (37)	0.155
Anticoagulation	2 (4)	3 (8)	0.646
Known onset	34 (65)	26 (68)	0.763
NIHSS score^b^	12.8 ± 6.8	15.76 ± 5.9	**0.035**
IV-tPA	32 (62)	19 (50)	0.275
MR/CT retaken prior to EVT (*N* = 50)	21 (42)	29 (58)	**0.002**
Time from stroke onset/recognition to puncture, min^a^	217 (170–298)	287 (220–371)	**0.010**
Time from stroke onset/recognition to reperfusion, min^a^	293 (224–342)	364 (290–494)	**0.004**
Door to puncture time, min^a^	55 (43–83)	60 (52–81)	0.153
Procedure time, min^a^	105 (78–135)	95 (71–138)	0.731
General anesthesia	40 (77)	21 (55)	**0.030**
Carotid pathology			
Dissection	14(27)	5 (13)	
Atherosclerosis	38 (73)	33 (87)	
Stent employed (*n* = 73)	42 (81)	31 (82)	0.923
Stent thrombosis	4 (10)	4 (13)	0.711
Intracranial lesion vs stent first			0.237
Perioperative antiplatelet therapy			0.116
Type of stent			0.847
Stented carotid pathology			0.060
mTICI ≥ 2b	50 (96)	32 (84)	**0.027**
HBC 1–2	21 (40)	23 (60)	**0.004**
HBC 3	6 (12)	8 (21)	0.099

Values are expressed as number (%) or ^a^median (IQR) or ^b^mean (SD)

*TIA* transient ischemic attack, *NIHSS* national institute of health stroke scale, *EVT* endovascular treatment, *IV-tPA* intravenous tissue-type plasminogen activator, *mTICI* modified thrombolysis in cerebral infarction, *HBC* Heidelberg bleeding classification score

## Discussion

Tandem cervical carotid occlusion did not lower the likelihood of good functional outcome compared to single occlusions. Factors associated with favorable outcome 90 days after treatment were lower age and NIHSS score, use of general anesthesia and higher mTICI score. Hemorrhage in the infarcted tissue after EVT and history of smoking were associated with poor outcome. Moreover, a history of diabetes and multiple stent retriever attempts were associated with an increased sICH risk.

The results in our study with similar outcomes in SO and TO may be related to our high-volume experience, the use of advanced imaging for patient selection, and the use of general anesthesia during stenting which can help secure good technical results. The ESCAPE-NA1 trial reported similar outcomes in TO compared to SO, showing in addition non inferiority of acute stenting in their study [14]. In earlier studies, emergent stenting of the cICA was associated with better outcomes [1, 3, 15]. Despite an increased risk of sICH in a recent meta-analysis, emergent stenting showed a trend towards lower mortality rate [5]. In our study, however, use of permanent stent was not associated with sICH. High proportion of cICA lesions treated with stent (81%) and consequently early antiplatelet therapy after intravenous thrombolysis did not increase the incidence of sICH. The rate of sICH in this study was comparable to the 8% rate of sICH earlier described in SO EVT [16]. Multiple attempts however, namely 3 or more stent retriever passes, have been suggested to increase the occurrence of sICH in SO EVT [17, 18]. Enomoto et al. reported that patients with more stent retriever passes were susceptible to subarachnoid hemorrhage [19]. Our study on TO demonstrate the same association between increased sICH risk and multiple retriever passes. Diabetes as a predictor for sICH in this study are in accordance with earlier studies reporting higher rates of hemorrhagic transformation or worse outcome in patients with diabetes mellitus in SO, though we did not find hyperglycemia to be associated with sICH as earlier reported [16, 19, 20].

Aside from sICH, stent thrombosis is also a main concern in treating TO. We found an 11% chance of stent thrombosis in the first 24 h. The use of dual layer stent may be preferred due to its better coverage of the atherosclerotic plaque or thrombus, however, stent thrombosis was reported to be more common [21]. This is the reason antiplatelet treatment should be tailored according to the stent used and the perceived risk of sICH. Eftifibatide may be an option and seem to be safe and effective in this study in accordance with an earlier study by Jost et al. [22]. Further studies on the drug’s safety and optimal dose in acute stroke treatment is needed.

The balance between sICH and stent thrombosis will always be a predicament in treating TO. Knowledge of possible predictors such as diabetes, number of stent passes and type of stent used, as exemplified in this study and earlier studies, may help guide clinicians and interventional neuroradiologist in their treatment strategies.

There is still equipoise in the use of GA vs CS [23–26]. However, in technical challenging procedures such as in TO achieving excellent technical results may be easier in GA. Although we did not find a difference in mTICI in this study, functional outcome at 90-day follow-up was better after GA.

The finding of worse outcome in the group with additional imaging compared to the patients who went directly to the angiography suite would naturally be interpreted as secondary to the extended time to recanalization. However, this is far more complex in clinical practice. Though direct to angio suite may shorten the door to groin puncture time, functional outcome did not differ in patients transferred from a local stroke unit to an EVT center [27]. Schaeffer et.al. demonstrated from a large registry that MRI before EVT in anterior SO and TO as well as posterior circulation were more likely to have better outcome. In our analysis, door to puncture time, which reflects the time used in repeat imaging, was not associated to 90-day mRS. The use of repeat imaging will always have its advantages and disadvantages which the treating physician would have to take into consideration.

As demonstrated in earlier studies age was directly associated with outcome. Although elderly patients may have less favorable outcome after EVT compared to younger adults, better results can still be achieved for selected elderly patients [28, 29]. Like earlier described with all types of AIS [30], history of smoking was shown to be associated with poor functional outcome in TO.

There are several limitations in our study. First, the retrospective and single center design as well as the small number of patients makes generalization of our findings limited. Pertinent variables such as actual blood pressure, post-stroke complications and management after discharge, which could affect outcome measures, was not included in the study. A full detailed comparison with SO was also not performed.

## Conclusions

In this study, thrombectomy with frequent use of emergency stenting of cICA in TO was associated with acceptable safety with low prevalence of sICH. Functional outcome was comparable to the technical less complex SO patients. Diabetes mellitus and increasing number of stent retriever attempts was associated with increased risk of sICH in TO. Higher TICI score and absence of hemorrhagic transformation predicted good functional outcome.

## Data Availability

Data gathered and used in this study is available upon reasonable request from the corresponding authors.

## Abbreviations

TO: Tandem occlusions
SO: Single occlusion
cICA: Cervical internal carotid artery
EVT: Endovascular treatment
sICH: Symptomatic intracerebral hemorrhage
MeVO: Medium vessel occlusion
LVO: Large vessel occlusion
GA: General anesthesia
CS: Conscious sedation
AIS: Acute ischemic stroke
CT: Computed tomography
MRI: Magnetic resonance image
DWI: Diffusion weighted imaging
AHA/ASA: American Heart Association and American Stroke Association
OSCAR: The Oslo acute reperfusion stroke study
NIHSS: National institute of health stroke scale
mRS: Modified Rankin scale
ASPECTS: Alberta stroke program early CT score
HBC: The Heidelberg bleeding classification
